# The soluble guanylate cyclase stimulator riociguat ameliorates pulmonary hypertension induced by hypoxia and SU5416 in rats

**DOI:** 10.1186/1471-2210-11-S1-P43

**Published:** 2011-08-01

**Authors:** Michaela Lang, Baktybek Kojonazarov, Norbert Weissmann, Friedrich Grimminger, Johannes-Peter Stasch, Werner Seeger, Hossein Ardeschir Ghofrani, Ralph Schermuly

**Affiliations:** 1 University of Giessen Lung Center, Giessen, Germany; 2 Bayer HealthCare, Cardiology Research, Wuppertal, Germany; 3 Department of Lung Development and Remodeling, Max-Planck Institute for Heart and Lung Research, Bad Nauheim, Germany

## Background

Plexiform lesions are a hallmark of severe pulmonary arterial hypertension (PAH) and despite the development of novel therapeutics approaches, the prognosis of patients with PAH is relatively poor. Riociguat is a stimulator of soluble guanylate cyclase (sGC) which has a dual mode of action: it sensitizes sGC to the body’s own NO and can also increase sGC activity in the absence of NO, causing vasorelaxation, anti-proliferation and anti-fibrotic effects. This is thought to be important because the NO levels in the pulmonary circulation are decreased in patients with PAH.

The aim of the study was to investigate the effects of riociguat as compared to the PDE5 inhibitor sildenafil on pulmonary vascular remodeling in severe experimental PH.

## Methods

Angioproliferative pulmonary hypertension was induced in rats by combined exposure to the vascular endothelial growth factor-receptor (VEGFr) antagonist SU5416 and hypoxia at 10%O2 (SU+HOX). Twenty-one days after SU+HOX, rats were randomized for oral treatment with riociguat (10 mg/kg BW/day), sildenafil (50 mg/kg BW/day) or vehicle for the next 14 days. Right ventricular internal diameter (RVID), tricuspid annular plane systolic excursion (TAPSE) and myocardial performance index (MPI) were measured by echocardiography (VEVO770, Visualsonics). Hemodynamic parameters were measured invasively at day 35. Pulmonary vascular remodeling was assessed by histomorphometric analysis of the degree of muscularization of small pulmonary arteries.

## Results

In rats with established PH, right ventricular systolic pressure (RVSP) was significantly decreased by treatment with riociguat to 73±4 mmHg (p=0.01) and sildenafil to 80±3 mmHg (p<0.05) as compared to placebo (89±3 mmHg). Riociguat, but not sildenafil markedly increased cardiac output (CO) to 60±5 ml/min (p<0.01) as compared with placebo (31±3 ml/min). Both, riociguat and sildenafil significantly decreased total pulmonary resistance index (p<0.001). Importantly, no significant difference in systemic arterial pressure (SAP) was detected between placebo and treated animals. These positive hemodynamic responses to riociguat and sildenafil were associated with partial reversal of structural remodeling of the lung vasculature. Additionally riociguat significantly reduced the proportion of occluded arteries and increased proportion of opened arteries and significantly decreased the neointima/media ratio. In addition, riociguat significantly decreased ratio of RV to LV+S to 0.55±0.29 as compared to placebo (0.76±0.02, p<0.001) and sildenafil treated animals (0.66±0.29, p<0.01). Moreover, riociguat improved RV function by normalization of TAPSE as compared to placebo (p<0.001) and sildenafil (p<0.05). Both compounds improved MPI measured by tissue Doppler imaging (p<0.001).

## Conclusion

We demonstrated that riociguat effectively suppresses pulmonary vascular remodeling and significantly improves RV function, measured via a range of invasive and non-invasive cardiopulmonary endpoints in an experimental model of severe PAH.

